# IMPACT OF BARIATRIC SURGERY ON THE INFLAMMATORY STATE BASED ON CPR VALUE

**DOI:** 10.1590/0102-672020180001e1402

**Published:** 2018-12-06

**Authors:** Renato MIGLIORE, João Kleber Almeida GENTILE, Fabiana Tornincasa FRANCA, Guilherme Tommasi KAPPAZ, Pedro Marcos Santinho BUENO-DE-SOUZA, José Cesar ASSEF

**Affiliations:** 1 Hospital of the Municipal Public Server, Technical Section of Digestive System Surgery, São Paulo, SP, Brazil.

**Keywords:** Gastric bypass, Inflammation, Anastomosis, Roux-en-Y, C-reactive protein, Derivação gástrica, Inflamação, Anastomose em Y-de-Roux, Proteína C-reativa

## Abstract

**Background:**

PCR (C-reactive protein), produced in the liver after stimuli of inflammatory mediators, is determined as a marker of inflammatory activity (adipocytokines) and is present within adipocyte cells; besides being an inflammatory product, many studies have shown to be a predictor of complications.

**Aim:**

To determine if the inflammatory state of the obese patient decreases after bariatric surgery, based on pre and post-operative PCR.

**Methods:**

A prospective, observational study in patients undergoing Roux-en-Y gastric by-pass surgery followed up for three months after surgery, with serum preoperative CRP in 30, 60 and 90 days after surgery.

**Results:**

A total of 19 patients, who had a mean CRP value before the surgical procedure of 0.80(±0.54) mg/dl, were followed, and when compared to the CRP with 30 days of surgery, they presented a significant increase to 2.68 mg/dl (p=0.012). When compared with the PCR of 60 days after the surgical procedure, it was also higher with the value of 3.32 mg/dl (p=0.27). However, at three months after surgery, the CRP showed a decrease when compared to the preoperative mark, with value of 0.45 mg/dl (p=0.0042).

**Conclusion:**

Roux-en-Y gastric bypass was able to decrease the chronic inflammation status of these patients, based on the value of CRP, with three months of surgery.

## RESUMO

**Racional:**

PCR (proteína C-reativa) produzida no fígado após estímulos de mediadores inflamatórios é determinada como um marcador de atividade inflamatória (adipocitocinas) e está presente nos adipócitos; além de ser um produto inflamatório, muitos estudos já mostraram ser ela um preditor de complicações.

**Objetivos:**

Determinar se o estado inflamatório do paciente obeso diminui após a realização de cirurgia bariátrica, com base na PCR do pré e pós-operatório.

**Métodos:**

Estudo prospectivo, observacional em pacientes submetidos à operação de by-pass gástrico em Y-de-Roux acompanhados por três meses após o procedimento com dosagem sérica da PCR no pré-operatório, 30, 60 e 90 dias após.

**Resultados:**

Foram acompanhados 19 pacientes, que apresentaram valor médio da PCR antes do procedimento cirúrgico de 0,80(±0,54) mg/dl e quando comparado à PCR com 30 dias de operados ela apresentou aumento significativo para 2,68 mg/dl (p=0,012). A análise da PCR de 60 dias após o procedimento cirúrgico mostrou-se maior também com o valor de 3,32 mg/dl (p=0,27). Entretanto no 3º mês após ela mostrou queda quando comparado ao pré-operatório (0,45 mg/dl (p=0,0042).

**Conclusão:**

O bypass gástrico em Y-de-Roux foi capaz de diminuir o estado de inflamação crônico desses pacientes com três meses de pós-operatório.

## INTRODUCTION

Obesity is a chronic disease characterized by excess body fat, which causes injury to the individual^1^. It has grown all over the world, both in developed and underdeveloped countries, with high costs in the treatment of its complications^18^ ^,^ ^27^ ^,^ ^30^. The World Health Organization estimates that each year 2.8 million people die as a result of being overweight or obese^32^. Currently in Brazil, the number of obese patients increased from 11.6% in 2006 to 18.9% in 2016^32^. The medical diagnosis of their complications, such as diabetes and hypertension, increased from 5.5% and 22.5% to 8.9% and 25.7% from 2006 to 2016 respectively^32^. Obesity is closely related to disease and metabolic disorders, the most common being insulin resistance, type 2 diabetes mellitus, hyperinsulinism, hypertension and dyslipidemia.

The obese patient is constantly associated with a state of chronic inflammation^18^ ^,^ ^27^. Inflammatory cytokines are not only produced in the inflammatory phase of a tissue lesion, but also by adipocytes^9^. It is believed that 70-80% of patients present remodeling of adipose tissue, both at structural and functional levels, causing chronic inflammatory reaction, low local reaction, called lipoinflammation, with leptin production and increased oxidative stress^12^ ^,^ ^14^. PCR (C-reactive protein), produced in the liver after stimuli of inflammatory mediators such as interleukin-6, tissue necrosis factors, interferon, among others, is determined as a marker of inflammatory activity (adipocytokines) and is present within adipocyte cells; besides an inflammatory product, many studies have shown to be a predictor of complications ^3^ ^,^ ^4^ ^,4,^ ^10^ ^,^ ^11^. PCR was first described in 1930 as a special protein found in the plasma of patients who were in the acute phase of pneumonia. Today it is considered a good method to evaluate inflammation and infection^13^ ^,^ ^14^. The increase in CRP levels is observed about two days after the onset of inflammation and because of its short shelf-life, is determined as a valuable marker for detecting postoperative complications^13^ ^,^ ^14^ ^,^ ^15^. In recent years, it has gained importance, also as a predictor of cardiac risk^16^. Ridker et al.^24^ showed that women with an average of 0.19 mg/dl had a relative risk of atherosclerosis of 2.1. It, greater than 0.3 mg/dl, is accepted as a cut-off for high cardiovascular risk^18^. Low risk is considered lower than 0.1 mg/dl and intermediate risk between 0.1-0.3 mg/dl^19^. Bochud et al.^5^ showed a positive correlation between BMI and CRP values ​​in obese women.

Bariatric surgery, or its new and more appropriate denomination metabolic surgery, propose a significant loss of fat mass in a short period of time, reducing the causes of morbidity and mortality caused by obesity^13^ ^,^ ^23^ ^,^ ^24^. It is able to reduce levels of leptin, inflammation and oxidative stress^10^ ^,^ ^25^.

Roux-en-Y gastric bypass (BGYR) is the most commonly performed technique today. It is presented as a good technique in weight loss and in the treatment of comorbidities, since it acts on food restriction and malabsorption, further reducing the secretion of ghrelin (oxygen hormone), insulin and leptin^3^ ^,^ ^24^. Increased production of GLP-1 and GLP-2, by not passing the food in the duodenum and its faster arrival in the distal ileum, act in this regulation of glycemic levels^3^ ^,^ ^8^.

Some studies show an association between CRP elevation and mortality predictor, or as a factor of postoperative complication in non-bariatric operations, such as cardiac, thoracic and others. However, few show if this chronic inflammatory state of the obese patient improves after the operation, based on the PCR.

The objective of this study was to determine if there is a decrease in the value of CRP in the bloodstream after BGYR, hypothesizing that bariatric surgery promotes improvement in the chronic inflammatory state in relation to the preoperative period. 

## METHODS

The project of this study was approved by the Ethics Committee of the Municipal Public Server Hospital (HSPM) with the number 58271316.8.0000.5442. Patients accepted and signed the term of free enlightenment with the possibility of leaving the research at any time without detriment or retaliation. It was prospective, observational, with 19 patients submitted to BGYR at the Municipal Public Server Hospital, São Paulo, SP, Brazil, from October 2016 to May 2017. All were between 18-70 years old, with or without comorbidities, with body mass index (BMI) between 35-50 kg/m². Those who had a diagnosis of pre-operative cholecystolithiasis were submitted to laparotomic cholecystectomy at the same surgical time. Patients with incisional hernias requiring correction at the same surgical time and those submitted to BGYR exclusively by the metabolic table (patients with BMI <35 kg/m^2^) were excluded.

In the preoperative period (approximately 1-2 months before the operation), ultra-sensitive PCR was requested by the immunoturbidimetry technique, always by the same laboratory, which presented as normal values ​​up to 5.0 mg/l in a fasting of at least 8 h. All were submitted to BGYR by the same team. After discharge, the same exams were requested at 30, 60 and 90 days.

### Statistical analysis

Statistical significance was p<0.05 and data were analyzed separately with other variables (comorbidities, BMI, percentage of weight loss, age and gender). In diabetic patients, the value of CRP was compared with values ​​of glycated hemoglobin (HbA1c) preoperatively. For the data analysis, the IBM SPSS Statistics 24® software was used and the paired Student’s t-test of two samples, Pearson’s correlation and Fisher’s test was used, evaluating BMI, preoperative PCR, postoperative with 30 , 60 and 90 days, with diabetic and non-diabetic patients and values ​​of glycated hemoglobin preoperatively.

## RESULTS

The study followed 19 patients for a period of 90 days after the date of the operation. It consisted of three men (15.7%) and 16 women (84.3%). The age ranged from 32-59 years (mean 45.15±8.38). From this sample, five had no comorbidities; 13 (68.4%) had diabetes or glucose intolerance and one had only arterial hypertension. Glycated hemoglobin (HbA1c) varied among diabetics from 5.3 to 9.9%. When the cutoff value for glycated hemoglobin ≥7.0 was applied as a decompensated diabetic patient, there were six (46.1%) of the 13 patients in this condition or with glucose intolerance using some oral hypoglycemic. BMI varied from 37.1 to 49.8 (mean 43.93±4.01) kg/m^2^. Four (23.5%) had preoperative grade II obesity and 15 (76.5%) had grade III. Weight ranged from 95-54 kg (mean 121.51±16.84, Table 1).

**TABLE 1:** Distribution of the study

		n/%	Mean±standard deviation
Gender	Fem	16 (84,3%)	-
	Male	3 (15,7%)	
Age	Min	32 anos	45,15 (±8,38) anos
	Max	59 anos	
Obesity	Grade II	4 (23,5%)	121,51kg (±16,84)
	Grade III	15 (76,5%)	
Comorbidities	Yes	5 (26,3%)	-
	No	14 (73,7%)	
Diabetes mellitus	HbA1c <7%	7 (53,9%)	5,8% (±0,37)
	HbA1c =7%	6 (46,1%)	7,65% (±0,94)

The value of CRP before the surgical procedure ranged from 0.2-1.98 mg/ (mean 0.80±0.54). When comparing the value of CRP before the surgical procedure between the diabetic and non-diabetic groups, it was found that in the non-diabetic group there was an average CRP value of 0.86 mg/l, whereas in the diabetic patients, 0.77 mg/l (p=0.78). When patients with glycated hemoglobin less than or equal to 7 mg/dl were separated from diabetics, a mean CRP of 0.83 mg/l was observed; among those with glycated hemoglobin greater than 7, there was an average of 0.69 mg/l (p=0.66). When comparing the preoperative CRP value between patients presenting grade II and grade III obesity, the mean CRP value was 0.56 and 0.86 mg/l, respectively (p=0.12 ).

At 30 days postoperatively, patients presented a mean weight loss of 10.52% (4.9-21.89±3.82). With weight loss greater than 10% in 30 days, it was possible to observe that 10/19 (52.6%) patients had a mean CRP value of 2.68±2.86 mg/l. When comparing preoperative values ​​with those of 30 days, mean increase from 0.8 to 2.68 mg/l (p=0.012, Pearson -0.034) was observed. At 60 days the mean weight loss was 15±3.96% (11.29-25.32) and the CRP value was between 0.2-43 mg/l (mean 3.32±9.70). However, when compared with preoperative CRP, it remained higher (Pearson -0.038), but without statistical significance (p=0.274). 

At 90 days the mean weight loss was 19.24 (14.28-37.01±5.49%). With goal loss greater than 20% in three months, it was possible to notice that 5/19 (26.3%) reached the goal. The mean PCR was 0.45 (1.3-0.02±0.31) mg/l. This association when calculated with Pearson’s correlation was positive (0.547, p=0.0042).

## DISCUSSION

Obese patients may present high CRP values ​​due to the chronic inflammatory condition developed by the increase of interleukin-6 and tumor necrosis factor in adipocytes, promoting the production of C-reactive protein by hepatocytes, inducing a state of chronic inflammation^3^ ^,^ ^14^ ^,^ ^23^. Due to the PCR/adipocyte ratio, it has been speculated that weight loss may decrease the chronic inflammatory state^21^. Increased inflammatory markers have been the focus of many studies, with emphasis on adipose tissue in obese patients as a causal factor of cardiovascular events^12^. In association with other studies, it was possible to show that bariatric surgery promotes a decrease in CRP, especially at three months^12^.

Differently from other studies, these patients did not present previous preoperative CRP greater than 3 mg/l which justified that, because they were obese, they would already have a high cardiovascular risk^3^ ^,^ ^19^. In the paper of Agrawal et al.^2^ the mean CRP value of obese patients was 1.12 mg/l, and few had a previous diagnosis higher than 3 mg/l, values ​​slightly above in the present study. Frask et al^11^ report data similar to the one found here with mean preoperative values ​​of 0.9±0.69 mg/l.

Bochud et al.^5^ in 2009 with 2836 women, studied the association between CRP values ​​and their relation with BMI and fat mass, concluding that there is a positive relation. The BMI presented association of 0.98 (p=0.004) while the fat mass of 2.07 (p=0.001). In this study there was no such comparison, since all were obese, but when separated by degree of obesity, the most obese had no higher CRP value. This relationship may not have been positive, since a single CRP result may not reflect the state of chronic inflammation, requiring examinations to be performed for each patient’s average^26^.

In this study, it was possible to initially find an increase in CRP in the first 30 days, and in the 90 days afterwards a mean value was lower than in the preoperative period, statistically significant, with weight loss already after 90 days of 20% in relation to the previous one. Borges et al.^6^ showed results very similar to that of this study with the beginning of the decrease in CRP only after three months and, associated with this decrease in CRP, evaluated that the amount of leukocytes and neutrophils also decreased, that is, decrease of biomarkers with the loss of weight. Studies indicate that the decrease in CRP is more accentuated by the decrease in waist circumference than in the loss of fat mass^6^.

Selvin et al^28^ found that at each weight loss of 1 kg a reduction in CRP of about 0.13 mg/l was promoted. They also reported that the largest decreases in CRP occurred in patients who presented more pronounced weight loss with bariatric surgery than with other procedures, such as liposuction.

In this study, weight loss was obtained in the 1^st^ month of the year^29^ ^,^ ^30^ ^,^ ^31^. At 90 days the result of the present study was similar to Ramos et al^23^ who studied 20 patients with BGYR, and obtained a mean weight loss 19.18% vs. 19.24% in this study.

Although bariatric surgery has been laparotomically performed, which is known to bring more postoperative pain, greater surgical trauma and longer surgical time, the elevation of CRP levels in the first 30 days cannot be associated with this state of tissue recovery by surgical trauma. Csendes et al^9^ analyzed the values ​​of the CRP in the immediate postoperative period and concluded that the CRP returns to the previous value of the operation already in the 5^th^ day and is also a good marker for the occurrence of fistulas. Such increase can be explained, since the rapid weight loss that occurs in the first 30 days promotes necroinflammatory activity in the liver, consequently increasing the CRP values^32^. Lins et al.^18^ associated high preoperative CRP levels with complications after BGYR.

No difference was found in the literature between the values ​​of CRP, statistically significant, in diabetic or non-diabetic patients. Evidence shows that type 2 diabetes mellitus corroborates chronic inflammation and insulin resistance^25^. Holdstock et al.^12^ reported a strong correlation between increased values ​​of CRP and diabetes or with fasting hyperinsulinemia. However, in the same study, they evaluated that all forms of weight loss, whether through physical activity, medication use or until bariatric surgery, showed a decrease in CRP values ​​compared to before the proposed therapy; however, some components of the insulin resistance syndrome did not improve. Other studies have shown that weight loss after gastroplasty progresses with decreased circulating levels of interleukin-6 and CRP in association with improved insulin resistance^32^.

## CONCLUSION

Roux-en-Y gastric bypass promoted a decrease in chronic inflammation of obese operated patients, evidenced by the decrease in CRP values ​​after three months of operation.

## References

[1] Andrade CGC, Lobo A (2014). Perda de peso no primeiro mês pós-gastroplastia seguindo evolução
de dieta com introdução de alimentos sólidos a partir da terceira
semana. ABCD Arq Bras Cir Dig.

[2] Agrawal V, Krause VR, Chengelis DL, Zalesin KC, Rocher LL, McCullough PA (2009). Relation between degree of weight loss after bariatric surgery
and reduction in albuminuria and c-reactive protein. Surg for Obes and R Diseases.

[3] Benchimol AK, Cardoso IS, Fandiño J, Bittar T, Freitas S, Coutinho WF (2007). Esteatoepatite Não Alcoólica Induzida por Rápida Perda de Peso em
Uso de Balão Intragástrico - Um Relato de Caso. Arq Bras Endocrinol Metab.

[4] Billeter AT, Vittas S, Israel B, Scheulen KM, Hidmark A, Fleming TH, Kopf S, Büchle MW, Stich M (2017). Gastric bypass simultaneously improves adipose tissue function
and insulin-dependent type 2 diabetes mellitus. Langenbecks Arch Surg.

[5] Bochud M, Marquant F, Vidal MPM, Vollenweider P, Beckmann JS, Mooser V, Paccaud F, Rousson V (2009). Association between C - reactive protein and Adiposity in
Women. J Clin Endocrinol Metab;.

[6] Borges RL, Ribeiro-Filho FF, Carvalho KMB, Zanella MT (2007). Impacto da Perda de Peso nas Adipocitocinas, na Proteína
C-Reativa e na Sensibilidade à Insulina em Mulheres Hipertensas com
Obesidade Central. Arq Bras Cardiol.

[7] Cazzo E, Gestic MA, Utrini MP, Pareja JC, CHaim EA, Geloneze B, Barreto MRL, Magro DO (2016). Correlação entre os níveis pré e pós-operatórios de GLP-1/GLP-2 e
a perda de peso após o bypass gástrico em y-de-roux um estudo
prospectivo. Arq Bras Cir Dig.

[8] CFM (2016). Resolução CFM Nº 2.131/2015.

[9] Csendes A, Burgos AM, Roizblatt D, Garay C, Bezama P (2009). Inflammatory Response Measured By Body Temperature, C-Reactive
Protein and White Blood Cell Count 1, 3, and 5 Days After Laparotomic or
Laparoscopic Gastric Bypass Surgery. Obes Surg.

[10] Flores MR, Salinas CA, Piché ME, Auclair A, Poirier P (2017). Effect of bariatric surgery on heart failure. Rev Expert Review of Cardiovascular Therapy.

[11] Frask A, Orlowski M, Wnukiewicz ND, Lech P, Gajewski K, Michalik M (2017). Clinical evaluation of C-reactive protein and procalcitonin for
the early detection of postoperative complications after laparoscopic sleeve
gastrectomy. Wideochir Inne Tech Maloinwazyjne.

[12] Holdstock C, Lind L, Engstrom BE, Ohrvall M, Sundbom M, Larsson A, Karlsson FA (2005). CRP reduction following gastric bypass surgery is most pronounced
in insulin-sensitive subjects. Int J of Obes.

[13] Illán-Gomez F, Gonzávez OM, Orea Sl, Alcaraz TMS, Aragón AA, Pascual DM, Pérez PM, Lozano AML (2012). Obesity and inflammation change in adiponectin, C-reactive
protein, tumour necrosis factor-alpha and interleukin-6 after bariatric
surgery. Obes Surg.

[14] Izaola O, Luis D, Sazoux I, Domingos JC, Vidal M (2015). Inflamación y obesidad (lipoinflamación). Nutr Hosp.

[15] Junges VM, Cavalheiro JM, Fam, Closs VE, Moraes JF, Gottlieb MG (2017). Impact of Roux-en-Y Gastric Bypass Surgery (RYGB) on metabolic
syndrome components and on the use of associated drugs in obese
patients. Arq Gastroenterol.

[16] Kopp HP, Kopp CW, Festa Um, Krzyzanowska K, Kriwanek S, Minar E, Roka R, Schernthaner L (2003). Impact of Weight Loss on Inflammatory Proteins and Their
Association with the Insulin Resistance Syndrome in Morbidly Obese
Patients. Arterioscler Thromb Vasc Biol.

[17] Lange LA, Carlson CS, Hindorff LA, Lange EM, Walston J, Durda JP, Cushman M, Bis JC, Zeng D, Lin D, Kuller LH, Nickerson DA, Psaty BM, Tracy RP, Reiner AP (2006). Association of polymorphisms in the CRP gene with circulating
C-reactive protein levels and cardiovascular events. JAMA.

[18] Lins DC, Campos JM, Paula OS, Galvão-Neto M, Pachu E, Cavalcanti N, Ferraz AAB (2015). Proteína c reativa em diabéticos antes do bypass gástrico como
possível marcador de complicação pós-operatória. Arq Bras Cir Dig.

[19] Melo LC, Mendonça da Silva MA, Calles ACN (2014). Obesidade e função pulmonar: uma revisão
sistemática. Einstein.

[20] Ministério da Saúde (2017). Obesidade cresce 60% em dez anos no Brasil.

[21] Nunes BK, Lacerda RA, Jardim JM (2011). Revisão sistemática e metanálise sobre o valor preditivo da
proteína C-reativa em infecção pós-operatória. Rev Esc Enferm USP.

[22] Pearson TA, Mensah GA, Alexander RW (2003). A statement for healthcare professionals from the centers for
disease control and prevention (CDC) and the American heart Association
(AHA). Circulation.

[23] Ramos AP, de Abreu MR, Vendramini RC, Brunetti IL, Pepato MT (2006). Decrease in Circulating Glucose, Insulin and Leptin Levels and
Improvement in Insulin Resistance at 1 and 3 Months after Gastric
Bypass. Obesity Surgery.

[24] Ridker PM, Hennekenens C, Rifai N (2000). C- reactive Protein and other markers of inflammation in the
prediction of cardivascular disease in women. New Engl J of Med.

[25] Santos J, Salgado P, Santos C, Mendes P, Saavedra J, Baldaque P, Monteiro G, Costa E (2014). Effect of bariatric surgery on weight loss, inflammation, iron
metabolism, and lipid profile. Scand J of Surg.

[26] Schmitz J, Evers N, Awazawa M, Nicholls HT, Brönneke HS, Dietrich A, Mauer J, Blüher M, Brüning JC (2016). Obesogenic memory can confer long-term increases in adipose
tissue but not liver inflammation and insulin resistance after weight
loss. Mol Metab.

[27] Segal A, Fandino J (2002). Indicações e contra-indicações para realização das operações
bariátricas. Rev Bras Psiquiatr.

[28] Selvin E, Paynter NP, Erlinger TP (2007). The Effect of Weight Loss on C-Reactive Protein. Arch Intern Med.

[29] Vieira RAL, Silva RA, Tomiya MTO, Lima DSC (2015). Efeito da cirurgia bariátrica sobre o perfil lipídico mais
aterogênico em curto prazo. Nutr Clín Diet Hosp.

[30] Vilarassa N, Vendrell J, Sánches-Santos R, Broch M, Megia A, Masdevall C, Gomez N, Soler J, Pujol J, Bettónica C, Aranda H, Gómes JM (2007). Effect of weight loss induced by gastric bypass on
proinflammatory interleukin-18, soluble tumour necrosis factor-a receptors,
c-reactive protein and adiponectin in morbidly obese
patients. Clin Endocrinol.

[31] Warschkow R, Tarantino I, Folie P, Beutner U, Schmied BM, Bisang P, Schultes B, Thurnheer M (2012). C-Reactive protein 2 days after laparoscopic gastric bypass
surgery reliably indicates leaks and moderately predicts
morbidity. J Gastrointest Surg.

[32] W.H. Organisation http://www.who.int/mediacentre/factsheets/fs311/en/index.html.

[33] Zagorski SM, Papa NN, Chung MH (2005). The effect of weight loss after gastric bypass on C-reactive
protein levels. Surgery for Obesity and Related Diseases.

